# Outcome of mitral valve repair or replacement for non-ischemic mitral regurgitation: a systematic review and meta-analysis

**DOI:** 10.1186/s13019-021-01563-2

**Published:** 2021-06-15

**Authors:** Qianqian Fan, Xiaoguang Li, Guilan Cao, Puliang Yu, Fengxiao Zhang

**Affiliations:** 1 Department of Cardiology, Union Hospital, Tongji Medical College, Huazhong University of Science and Technology, Hubei Province, 430022 China; 2 Department of Nephrology, Wuhan HanKou Hospital, Hubei Province, 430012 China; 3 Department of Cardiology, Hubei No.3, People’s Hospital of Jianghan University, Wuhan, 43003 China; 4 Key Laboratory of Metallurgical Equipment and Control Technology, Wuhan University of Science and Technology, Hubei Province, 430081 China

**Keywords:** Non-ischemic mitral regurgitation, Mitral valve repair, Mitral valve replacement, Meta-analysis

## Abstract

**Background:**

Mitral regurgitation (MR) is a rather common valvular heart disease. The aim of this systematic review and meta-analysis was to compare the outcomes, and complications of mitral valve (MV) replacement with surgical MV repair of non-ischemic MR (NIMR)

**Methods:**

MEDLINE, EMBASE, and the Cochrane Central Register of Controlled Trials were searched until October, 2020. Studies were eligible for inclusion if they included patients with MR and reported early (30-day or in-hospital) or late all-cause mortality. For each study, data on all-cause mortality and incidence of reoperation and operative complications in both groups were used to generate odds ratios (ORs) or hazard ratios (HRs). This study is registered with PROSPERO, CRD42018089608.

**Results:**

The literature search yielded 4834 studies, of which 20 studies, including a total of 21,898 patients with NIMR, were included. The pooled analysis showed that lower age, less female inclusion and incident of hypertension, significantly higher rates of diabetes and atrial fibrillation in the MV replacement group than MV repair group. No significant differences in the rates of pre-operative left ventricle ejection fraction (LVEF) and heart failure were observed between groups. The number of patients in the MV repair group was lower than in the MV replacement group. We found that there were significantly increased risks of mortality associated with replacement of MR. Moreover, the rate of re-operation and post-operative MR in the MV repair group was lower than in the MV replacement group.

**Conclusions:**

In patients with NIMR, MV repair achieves higher survival and leads to fewer complications than surgical MV replacement. In light of these results, we suggest that MV repair surgery should be a priority for NIMR patients.

**Supplementary Information:**

The online version contains supplementary material available at 10.1186/s13019-021-01563-2.

## Introduction

Mitral regurgitation (MR) is a rather common form of mitral valve (MV) dysfunction, occurring in approximately 10% of the population [1]. Normal mitral closure depends on the integrity and normal function of five components: the leaflets, annulus, tendons, papillary muscles, and left ventricle. Abnormalities in the structure and function of any one of these five components can lead to MR. Patients with mild MR have only occasional exertional dyspnea, and can be asymptomatic. However, acute severe MR (e.g., papillary muscle rupture) can lead to acute left heart failure or even cardiac death.

Severe MR often requires surgical correction, which is useful for improving the prognosis and quality of life of the patients. Traditionally, MV surgery for mitral regurgitation consists mainly of MV replacement and MV repair. According to several lines of evidence from comparative studies, MV repair, which has been increasingly more often performed since 1980s, is preferred over MV replacement [2], and currently, more and more patients benefit from successful valve repair operations. However, whether to replace or repair severe MR, remains a controversial topic. Previously, the majority of surgeons believed that MV repair was of low surgical risk, with few near and long-term adverse events, and offered optimal survival environment and quality of life; therefore, and given its ability to preserve the heart’s natural structure intact and achieve optimal cardiac functional reserve, it became the preferred surgical treatment modality for mitral valve disease. However, there are still questions about the durability of mitral valve repair due to conceptual, technical, and real-world limitations. Moreover, recent data comparing MV repair and replacement failed to demonstrate superiority of MR repair in ischemic MR patients [3]. Altogether, these findings challenge the benefits and the superiority of MV repair over replacement.

The etiology of MR is heterogeneous and may lead to outcome bias after valve surgery. There are many causes of MR, including rheumatic fever, coronary heart disease, congenital valve malformations, and mitral valve degeneration. In addition, ischemic mitral valve insufficiency is a complex condition, with several associated issues as the need for revascularization, susceptibility to comorbid ventricular wall tumors or myocardial scars, and severe left heart dysfunction [4]. In this study, we selected studies that included patients with non-ischemic mitral valve insufficiency. We performed a systematic review and a meta-analysis comparing clinical results of isolated MV surgery in patients with non-ischemic MR (NIMR).

## Methods

### Standard of systematic reviews

This study is designed and performed according to the “Transparent reporting of systematic reviews and meta-analyses” (PRISMA) guidelines (see Supplemental material). This systematic review and meta-analysis have been registered with the International Prospective Register of Systematic Reviews (PROSPERO, ID: CRD42018089608).

### Search strategy

We systematically searched for studies, including randomized controlled trials (RCTs) and observational comparative studies, of surgical MV replacement versus surgical MV repair that enrolled patients with NIMR. Databases including MEDLINE, EMBASE, and the Cochrane Central Register of Controlled Trials were searched from inception to October, 2020 using Web-based search engines (PubMed and OVID). The search strategies employed a number of free-text keywords as well as controlled vocabulary terms (see Appendix A for actual search strategies).

### Selection criteria and data abstraction

Studies were considered for inclusion if they met the following criteria: the design was a RCT or observational comparative study and the study population included patients with NIMR; patients were assigned to MV replacement versus MV repair; and main outcomes reported included early (30-day or in-hospital) or late (≥6-month including early) all-cause mortality. Data regarding detailed inclusion criteria, baseline patient profiles, duration of follow-up, all-cause mortality, and etiology of MR were abstracted (as available) from each individual study.

### Data extraction and risk of bias assessment

One investigator (F-X.Z.) used a standardized form to extract the following relevant data and another investigator (P-L.Y.) independently confirmed their accuracy. Disagreement was resolved by discussion with another investigator (QQ. F.). The study quality was assessed using a previously proposed scale (newcastle-ottawa scale) [5, 6]. A greater overall score indicated a lower risk of bias; a score of 5 or less (of 9) suggested a high risk of bias. Risk of bias also was evaluated independently by two authors(G-L.C. and QQ. F.).

### Statistical analysis

We conducted a meta-analysis of summary statistics from the individual studies. We generated mean differences (MDs) and 95% confidence intervals (CIs) using means (with standard deviations) of age, left ventricle ejection fraction (LVEF) in both the MV replacement and MV repair groups, as well as risk (rate) differences (RDs) using rates of female patients, patients with diabetes, hypertension, heart failure, and New York Heart Association (NYHA) functional class (≥III) in both groups. HR and 95% CIs were used to estimate the effect of the intervention on patient survival.

If HR and 95% CI values were not available, Kaplan-Meier curves were read using Engauge Digitizer version 4.1, and the survival data were then entered in the spreadsheet based on previous work by Liu et al. [7]. Summary HRs (for total survival) and the summary ORs (for 30-day survival and re-operation) were directly abstracted from each study. For a study without available HRs, an HR was calculated based on the Kaplan–Meier curve [8] or summary data using the methods by Parmar et al. [9]. Heterogeneity among the included studies was assessed by Cochran’s Q statistics and by I^2^ test. If significant heterogeneity was obtained on analysis, we used reported cut-off values (> 50% mild, 51 to 75% moderate, and > 75% significant) and the random-effects model; otherwise, we used the fixed model. Publication bias was assessed visually on a funnel plot and statistically using a mixed-effects Egger’s test. All analyses were conducted using Review Manager version 5.3 [REFERENCE or weblink].

## Results

### Search results

**Fig. 1:**
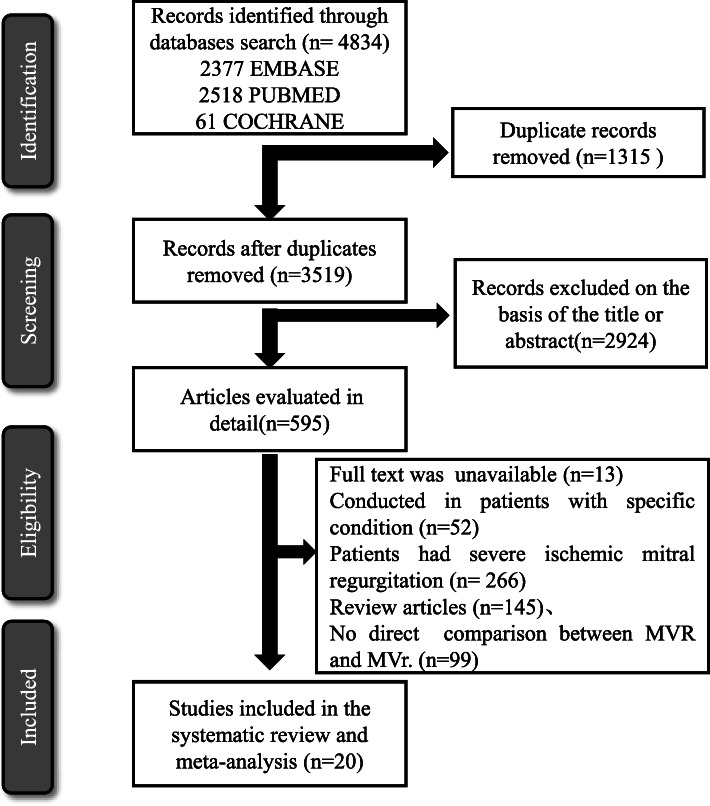
Flowchart depicting study selection for meta-analysis

Our search identified 4834 studies and 20, enrolling a total of 21,898 patients with NIMR, were included in the final analysis (Table S1 & Fig. 1) [2, 10–26]. Four studies were retrospective [11–13, 21], two were case-control studies [15, 20], and 14 studies were prospective cohort studies. Two studies exclusively enrolled hypertrophic obstructive cardiomyopathy patients with MR [13, 14] and two studies included only Marfan syndrome cases with MR [15, 16], whereas four studies excluded ischemic MR [10, 11, 23, 24]. Eleven studies enrolled patients with degenerative MR. The mean follow-up duration was from 30 days [27] to more than 10 years [13]. When available, prevalence of risk factors of surgery for each study is given in Table S2. These risk factors included heart failure (New York Heart Association (NYHA) class III or IV, low pre-operative LVEF), and prior history of hypertension, diabetes, or atrial fibrillation.

### Quality assessment

The quality assessment of the 20 included studies is shown in Table S3. According to the Newcastle-Ottawa Scale to assess the risk of bias in these studies, 17 studies scored between 6 and 9, indicating high methodologic quality. The remaining three studies scored …

### Patient profiles

#### Age

Fifteen studies provided exact values for mean age of two groups. The average age in most of the studies was between 50 and 78 years (Table S1). There was a study reported using younger patients [15]; while another study used very elderly subjects [12]. A pooled analysis demonstrated that patients of MV repair group were slightly younger than the ones in the MV replacement group (1.92; [1.74, 2.11]; p = < 0.0001; Supplemental Fig. S1).

#### Gender

Fourteen studies provided exact numbers of women for two groups. Most studies included more male patients than female. The percentage of women included was substantially higher than men only in two studies (Table S2) [14, 27]. The percentage of women included was lower in the MV repair group than in the MV replacement group (1.37; [1.28, 1.47]; *p* < 0.0001; Supplemental Fig. S2).

#### Pre-operative cardiac function

Only six of the 20 studies provided precise values of pre-operative LVEF for two groups. Eleven studies supplied the numbers of NIMR patients with NYHA functional class (≥III), and three studies disclosed the numbers of NIMR patients with heart failure. Our results demonstrate that there was no difference in number of pre-operative LVEF (0.03, [− 0.16, 0.23]; *p* = 0.01, Supplemental Fig. S3A), or heart failure (1.02, [0.77, 1.35]; *p* = 0.87, Supplemental Fig. S3C) between the two groups, but the number of individuals of NYHA functional class (≥III) in the MV repair group was slightly lower than in the MV replacement group (1.19, [1.01, 1.41]; *p* = 0.0009, Supplemental Fig. S3B).

#### Risk factors

Only five of the 20 studies provided exact numbers of patients with diabetes for the two groups. Six studies disclosed the numbers of NIMR patients with hypertension, and 11 studies disclosed the numbers of MR patients with atrial fibrillation (AF). The rate of diabetes (3.04, [2.03, 4.54]; *p* = 0.003, Supplemental Fig. S4A), hypertension (1.45, [1.33, 1.58]; *p* < 0.00001, Supplemental Fig. S4B) and AF (1.31, [1.22, 1.41]; *p* = 0.50, Supplemental Fig. S4C) were lower in the MV repair group than in the MV replacement group.

### Outcomes

#### Early mortality

**Fig. 2:**
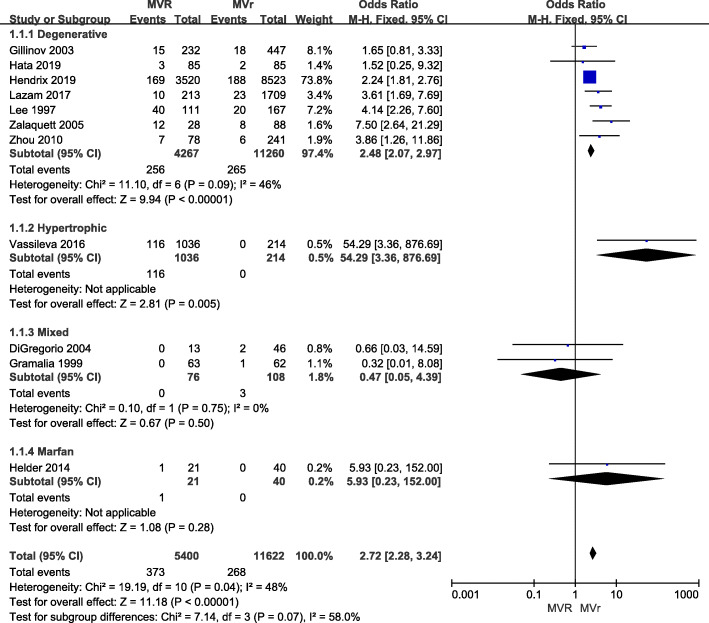
Forest plot of OR of early mortality for MV REPAIR versus MV repair

OR for early mortality was available for 12 articles. However, these studies were not fully consistent in their definition of early mortality. For the purpose of the current analysis, early outcomes labeled ‘early mortality’ (*n* = 1), ‘hospital mortality’ (*n* = 2), ‘operative mortality’ (*n* = 5), and ‘30-day mortality’ (*n* = 4) were combined under the ‘early mortality’ label. There was no early mortality among the patients in one of the studies [16]. Unadjusted ORs for early mortality were available for 11 studies, two of which suggested a significantly lower early-mortality rate in the MV repair group than in the MV replacement group [12, 23]. The pooled analysis of these studies showed a summary OR of 2.72 ([2.28, 3.24]; *p* < 0.00001), suggesting that people in the MV replacement group have a significantly increased risk of early mortality compared to people in the MV repair group (Fig. 2). Studies included subgroups according to the etiology of MR, and seven of these studies specifically focused on degenerative MR. The heterogeneity test was not statistically significant (*p* = 0.09, I^2^ < 50%) for the degenerative subgroup, nor for the other included studies, suggesting no clear evidence of a major discrepancy among the ORs for the studies analyzed.

#### Late survival

**Fig. 3:**
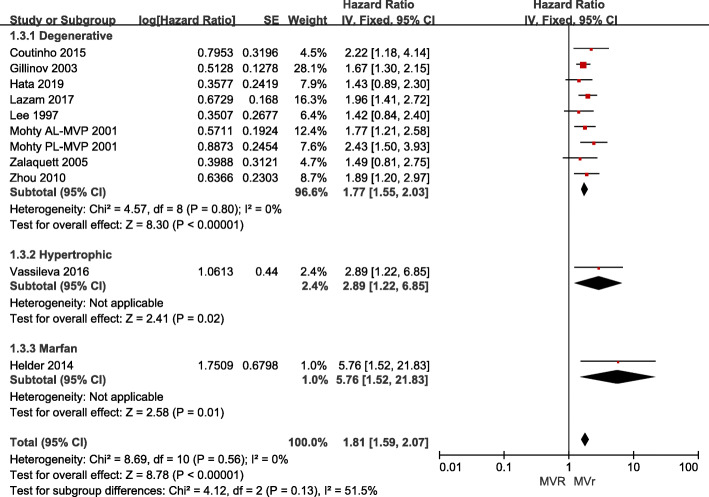
Forest plot of HR of late mortality for MV REPAIR versus MV repair

HR for late mortality was available in 10 articles, four of which provided the adjusted HR [2, 18, 24, 28]. HR for 20 years survival was provided in two studies [2, 18], one study provided 6 years survival data [25], one study provided 5 years survival data [21], while 10 year survival HR was available in five articles [13, 15, 19, 24, 26, 28]. A pooled analysis indicated statistically higher late-mortality rate in the MV replacement group than in the MV repair group (1.81, [1.59, 2.07]; *p* = 0.56, Fig. 3). Studies included subgroups according to the etiology of MR, and seven of these studies focused on degenerative MR. The heterogeneity test was not statistically significant (*p* > 0.10, I^2^ < 50%) for the degenerative subgroup, nor for the other included studies, suggesting no clear evidence of a major discrepancy among the hazard ratios for the studies analyzed.

#### Reoperation

**Fig. 4:**
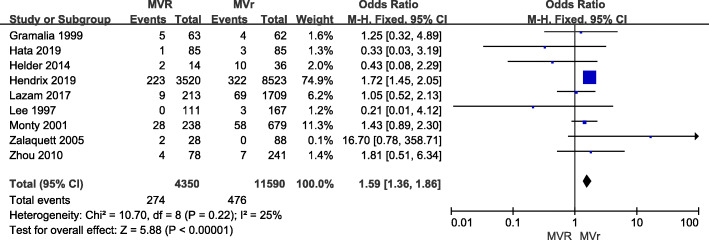
Forest plot of OR of re-operation for MV REPAIR versus MV repair

Nine articles provided information to allow determination of the re-operative OR for MV replacement relative to repair. Results demonstrated that the risk of reoperation is higher in MV replacement group than in MV repair group (1.59; [1.36, 1.86]; *p* = 0.22; Fig. 4).

#### Complications

Major complications of MV surgery were post-operative MR (including residual or recurrent MR), thromboembolism, heart failure, infective endocarditis. No sufficient information for determining the hazard ratio for development of these complications was provided in the studies.

Data on post-operative severe MR were analyzed. Only four articles provided exact numbers of post-operative MR in the two groups. Our results suggest that the incidence rate of post-operative severe MR was lower in the MV repair group than in the MV replacement group (1.43; [1.13, 1.82]; *p* = 0.01; Fig. S5). Additional post-operative complications were studied in only two articles [23, 25], both of which suggested beneficial effects from repair compared to replacement for thromboembolism and heart failure. One study [23] demonstrated that more patients had infective endocarditis in the MV replacement group than in the MV repair group, while another study [25] suggested a comparable incidence rate of infective endocarditis in the two groups.

### Publication bias

**Fig. 5:**
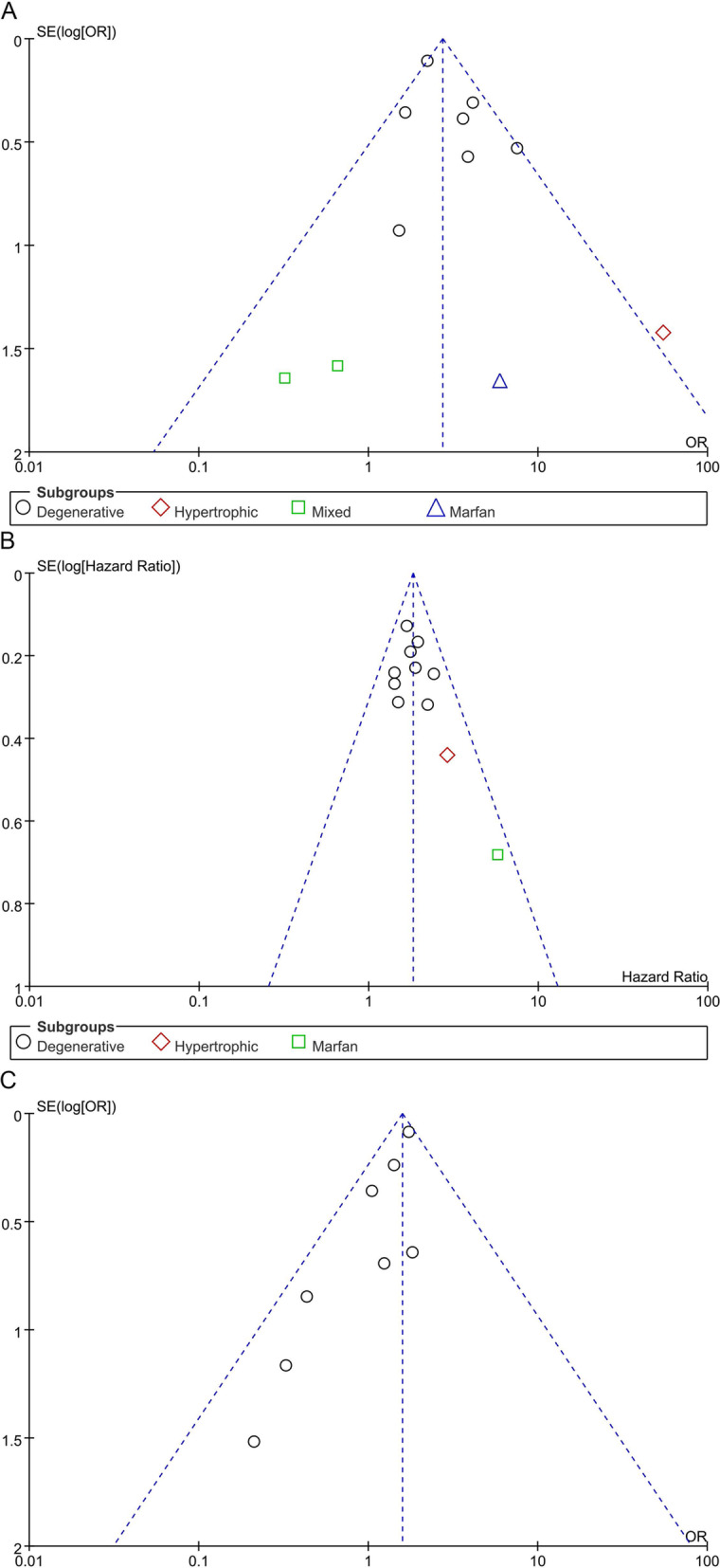
Funnel plot of early (**A**) and late (**B**) mortality

Inspection of the funnel plot (Fig. 5) did not show significant asymmetry for early and late mortality, suggesting that publication bias in unlikely to have influenced the results.

## Discussion

The current meta-analysis is an attempt to pool basic available information on the outcome experience of the two treatment methods for NIMR, specifically defined as MV repair and MV replacement. We included 20 studies, comprising a total of 21,898 patients. We demonstrate a statistically significant early and late survival benefit for patients who have undergone MV repair vs MV replacement. MV repair also led to a lower rate of re-operation and post-operative MR than surgical MV replacement. However, there was insufficient information for determining the hazard ratio of other surgery complications. In line with our findings, a number of articles previously indicated that the post-operative risk of thromboembolism and heart failure was lower for MV repair compared to MV replacement [23, 25].

It is generally regarded that the outcome after MV repair is better than that after MV replacement, and indeed valve repair operation has become the first choice in patients with MV diseases [29, 30]. In agreement with previously published data, we found that early and late mortality rates of NIMR in the MV repair subgroup significantly favored the repair group. Many experts believe that mitral valve replacement is superior to mitral valve replacement in the recovery of cardiac function due to the reasons of retaining chordae tendinae. However, the present findings should be interpreted with caution, because of higher risk profiles (high rates of diabetes and AF) in the MV repair than in the surgical MV replacement group. Previous studies suggested that diabetes and AF are associated with significantly worse outcomes after valve operations [31, 32]. Rates of diabetes and AF can also affect the heart function of NIMR patients. Even so, higher survival rates in the MV repair group than in surgical MV replacement for NIMR suggest survival benefits after MV repair, despite higher risk profiles of the patients included in this group.

Different etiologies can affect different mitral apparatus and lead to various pathologic alterations and outcomes [33]. Common causes of MR are congenital cleft MV, rheumatic heart disease, MV prolapse, infective endocarditis, rupture of chordae tendineae, Marfan syndrome, hypertrophic cardiomyopathy, endocardial fibrosis, and ischemic heart disease [34]. This systematic review summarizes characteristics and outcomes in patients with degenerative, hypertrophic, Marfan and mixed mitral valve regurgitation. Most of the include studies enrolled a cohort of patients with degenerative subjects. MV repair exhibited a survival advantage in in comparison to MV replacement. More articles are needed for a comprehensive analyzes of benefits of MV repair in different conditions.

In this systematic review, and meta-analysis we found that the risk of re-operation was higher in the MV replacement than in the repair group. Theoretically, patients who had unsuccessful valve repair should have worse outcomes than those who underwent direct mitral valve replacement due to the increased complexity of repair surgery and longer cardiopulmonary bypass time [35, 36]. Knowledge of the risk factors for the failure of MV is critical to help reduce the rates of reoperation. Reasons for re-operation included technical mistakes and valve-related causes (e.g. infection, progression of disease, and thrombosis). The main reason of re-operation in patients with NIMR was post-operative MR, including residual or recurrent MR [37]. In this review and meta-analysis, the incidence rate of post-operative MR was higher in MV replacement than in the MV repair group. The main mechanism of recurrent regurgitation after MV repair is progressive degeneration. Surgical repair procedures differed in some details, according to method of annuloplasty (suture vs. ring), type of ring annuloplasty (flexible or rigid), and chordal modification (transposition, shortening and replacement) [29]. Variations could help to improve the effect of surgery and minimize valvular failure. Zhou et al. identified the following factors as having an influence on the durability of repair for degenerative mitral valve disease: age < 60 years, ring size (mm)/body surface area (m^2^) ≥ 19.0, absence of a prosthetic ring and residual mitral regurgitation (≥ 1/4) at the end of surgery [21]. The selection of a surgical procedure (repair vs. replacement) is a multifactorial process that involves the surgeon, the patient and institution. The findings provided in this systematic review and meta-analysis help provide guidance regarding the best type of approach for each patient.

### Limitations

The results are based on data pooled from retrospective, case-control and prospective cohort studies, but no RCT studies were included. The study by Hendrix and colleagues [27], which included 12,043 subjects, carried disproportionate weight in the analysis of the results. Moreover, most studies collected subjects with degenerative NIMR. Therefore, biases are likely to have occurred due to the different type, weight and etiology of the studies included, and for this reason the results presented here should be interpreted with caution. In addition, more studies are needed to supply sufficient information to quantify the effect of etiology and the HR for surgery complications.

## Conclusions

In conclusion, repair surgery for NIMR was associated with a lower 30-day mortality and higher survival than replacement despite risk profiles (higher rate of diabetes and AF in the repair group). We observed lower rates of re-operation and post-operative MR in the repair group of non-ischemic mitral valve. However, etiology-related differences in the risk of re-operation remain uncertain and further studies and trials are needed. The results of this systematic review and meta-analysis can provide guidance for doctors and patients regarding the choice of mitral valve surgery and help to predict outcomes of NIMR patients.

## Supplementary Information

**Additional file 1: Table S1** Demographic information on included studies. **Table S2** Risk factors of patients undergoing mitral valve versus replacement. **Table S3**. Quality ratings for the cohort studies included on the basis of Newcastle-Ottawa quality assessment scale.

**Additional file 2.**

**Additional file 3.**

**Additional file 4.**

**Additional file 5.**

**Additional file 6.**

## Data Availability

The datasets used and analyzed during the current study are available from the corresponding author on reasonable request.

## Abbreviations

CIs: Confidence intervals
HRs: Hazard ratios
LVEF: Left ventricle ejection fraction
MR: Mitral regurgitation
MV: Mitral valve
NIMR: Non-ischemic MR
MDs: Mean differences
NYHA: New York Heart Association
RCTs: Randomized controlled trials
RDs: Differences
ORs: Odds ratios
