# Supplementary material for: Outcome of mitral valve repair or replacement for non-ischemic mitral regurgitation: a systematic review and meta-analysis
Source: J Cardiothorac Surg. 2021 Jun 15;16:175. doi: 10.1186/s13019-021-01563-2 (PMC8207733; doi:10.1186/s13019-021-01563-2)
Supplement: Supplementary file 1 — Additional file 1: Table S1 Demographic information on included studies. Table S2 Risk factors of patients undergoing mitral valve versus replacement. Table S3. Quality ratings for the cohort studies included on the basis of Newcastle-Ottawa quality assessment scale. [file 13019_2021_1563_MOESM1_ESM.doc]

Table S1 Demographic information on included studies。

| Study | Publication  year | Country | Subjects | | Mean age | | Male/female | | | Follow-up  (years) | | | | Etiology |
| --- | --- | --- | --- | --- | --- | --- | --- | --- | --- | --- | --- | --- | --- | --- |
|  |  |  | MV replacement | MV repair | MV replacement | MV repair | MV replacement | MV repair | | MV replacement | | MV repair | |  |
| Bozbuga et al[1] | 2003 | Turkey | 3 | 3 | NR | NR | NR | NR | | 4.51 | | | | Marfan |
| Coutinho et al[2] | 2015 | Portugal | 26 | 475 | 65.0±13.6 | 62.2±15.4 | 21/5 | 332/143 | | 7.9 ± 5.4 | | | | Degenerative |
| DiGregorio et al[3] | 2004 | USA | 13 | 46 | 82±2 | 82±2 | 7/6 | 18/28 | | 5.1 ± 2.2 | | | 5.8±3 | Mixed |
| Gillinov et al[4] | 2003 | USA | 232 | 447 | 67± 9.4 | 67± 9.1 | 160/73 | 336/111 | | 5.2 ± 3.6 | | | | Degenerative |
| Gramalia et al[5] | 1999 | Italy | 63 | 62 | 55±13 | 55±14 | 40/23 | 37/25 | | NR | | NR | | Mixed |
| Grapsa et al[6] | 2013 | Korea | 34 | 9 | 59.5 ± 15.4 | NR | NR | NR | | NR | | Degenerative | |  |
| Helder et al[7] | 2014 | USA | 21 | 40 | 31±19 | 40±18 | 7/14 | 27/13 | | 12±8 | | 7±5 | | Marfan |
| Hong et al[8] | 2016 | USA | 41 | 133 | 59.1±14.3 | 24/19 | 76/57 | | | >10 | | | | Hypertrophic |
| Lazam et al[9] | 2017 | Belgium | 213 | 1709 | 67±11 | 65±12 | 141/72 | 1265/444 | | 4.4 to 18.1 | | | | Degenerative |
| Lee et al[10] | 1997 | UK | 111 | 167 | 64.8±9.7 | 66±9.2 | 61/50 | 114/53 | | 3.23±2.23 | | | | Degenerative |
| Mohty et al[11] | 2001 | USA | 238 | 679 | 65±13 | | 624/293 | | | 7.7±4.1 | | | | Degenerative |
| Pandis et al[12] | 2011 | United Kingdom | 19 | 21 | 59.5±15.4 | | NR | NR | | 0.5 | | | | Degenerative |
| Shaﬁi et al[13] | 2012 | USA | 171 | 2607 | 69±13 | 56±13 | 77/94 | 1731/876 | | NR | | | NR | Degenerative |
| Sénéchal et al[14] | 2013 | Canada | 30 | 42 | 60±13 | | 53/72 | | | 0.69 to 3.64 | | | | Mixed |
| Tourneau et al[15] | 2000 | USA | 16 | 24 | 65±7 | 55±13 | 8/8 | | 8/16 | within one year | | | | Mixed |
| Vassileva et al[16] | 2011 | USA | 1036 | 219 | 63.3±13.3 | 60.0±16.3 | 384/652 | | 79/140 | 3 | | | | Hypertrophic |
| Zalaquett et al[17] | 2005 | Chile | 28 | 88 | 61.3±14.6 | 59.9±14.8 | 19/9 | | 48/40 | 6.725±3.81 | 4.47±2.72 | | | Degenerative |
| Zhou et al[18] | 2010 | USA | 78 | 241 | 69.8± 10.5 | 67.3± 10.6 | 45/33 | | 123/121 | 6.1 ±3.3 | 5.2 ±3.7 | | | Degenerative |
| Hendrix et al[19] | 2019 | USA | 3520 | 8523 | 78±4.9 | 76±4.7 | 1424/2096 | | 4163/4360 | 30days | 30days | | | Degenerative |
| Hata et al [20] | 2019 | Germany | 85 | 85 | 64.5 ± 13.3 | 64.3 ± 11.2 | 52/32 | | 52/32 | 0–98 months | 1–98 months | | | Degenerative |

Table S2 Risk factors of patients undergoing mitral valve versus replacement

| Study | Pre-operative LVEF (%) | | Post-operative LVEF (%) | | Pre-operative NYHA class III–IV (%) | | Congestive heart failure (%) | | diabetes (%) | | Hypertension (%) | | Pre-operative AF(%) | | Post-operative AF(%) | |
| --- | --- | --- | --- | --- | --- | --- | --- | --- | --- | --- | --- | --- | --- | --- | --- | --- |
|  | MV replacement | MV repair | MV replacement | MV repair | MV replacement | MV repair | MV replacement | MV repair | MV replacement | MV repair | MV replacement | MV repair | MV replacement | MV repair | MV replacement | MV repair |
| Bozbuga et al[1] | 42 | 40 | 50 | 52 | 66.7 | 100 | NR | NR | NR | NR | NR | NR | NR | NR | 66.7 | 0 |
| Coutinho et al[2] | 61.1±13.0 | 63.5±10.9 | NR | NR | 84.6 | 48.6 | 23.1 | 6.9 | 28.3 | | 26.9 | 28.4 | 45.5 | 33.5 | 45.5 | 35.5 |
| DiGregorio et al[3] | 63±12 | 63±9 | NR | NR | 77 | 80 | NR | NR | 61.53 | 4.35 | NR | NR | 46 | 52 | NR | NR |
| Gillinov et al[4] | NR | NR | NR | NR | 35 | 38 | NR | NR | 14 | 10 | 58 | 55 | 25 | 21 | NR | NR |
| Gramalia et al[5] | NR | NR | NR | NR | 100 | 88.7 | NR | NR | NR | NR | NR | NR | 30.2 | 35.5 | NR | NR |
| Grapsa et al[6] | 65.3±9.3 | | 42±7.6 | | 72.5 | | NR | NR | NR | NR | NR | NR | 62.5 | | 5 | |
| Helder et al[7] | 56±8 | 60±7 | NR | NR | 90 | 98 | NR | NR | NR | NR | NR | NR | NR | NR | 4.9 | |
| Hong et al[8] | NR | NR | NR | NR | 84 | | NR | NR | 7 | | NR | NR | NR | NR | NR | NR |
| Lazam et al[9] | 66± 9 | 64±10 | NR | NR | 39 | 34 | NR | NR | 3.75 | 0.47 | 20.2 | 2.3 | 32 | 28 | NR | NR |
| Lee et al[10] | NR | NR | NR | NR | 64.5 | 71.9 | NR | NR | NR | NR | NR | NR | 61.8 | 51.5 | NR | NR |
| Mohty et al[11] | 62±10 | | NR | NR | 51 | | NR | NR | NR | NR | NR | NR | 42 | | NR | NR |
| Pandis et al[12] | 62.3±13.3 | 58.1 ±10.8 | NR | NR | 72.5 | | NR | NR | NR | NR | NR | NR | 62.5 | | 5 | |
| Shaﬁi et al[13] | NR | NR | 56 ± 8.0 | 58±7.4 | NR | NR | 51 | 21 | NR | NR | NR | NR | 33 | 11 | NR | NR |
| Sénéchal et al[14] | 55±8 | | NR | NR | NR | NR | NR | NR | NR | NR | NR | NR | NR | NR | NR | NR |
| Tourneau et al[15] | 64.1 | 64.3 | 57.4 | 61.5 | NR | NR | NR | NR | NR | NR | NR | NR | 38 | 17 | NR | NR |
| Vassileva et al[16] | NR | NR | NR | NR | NR | NR | 33.6 | 37.8 | NR | NR | NR | NR | NR | NR | NR | NR |
| Zalaquett et al[17] | NR | NR | NR | NR | 50 | 63 | NR | NR | NR | NR | NR | NR | NR | NR | NR | NR |
| Zhou et al[18] | NR | NR | NR | NR | 47.4 | 28.6 | NR | NR | 30.8 | 32.4 | 2. 6 | 1.2 | 23.1 | 24.9 | NR | NR |
| Hendrix et al[19] | 60±5 | 60±5 | NR | NR | NR | NR | NR | NR | NR | NR | 80 | 74 | 52 | 45 | 32 | 32 |
| Hata et al [20] | NR | NR | 66.1 ± 8.9 | 65.4 ± 8.7 | 54.1 | 45.9 | NR | NR | 4.7 | 9.4 | 68.2 | 67.1 | 37.6 | 29.4 | 21.1 | 18.8 |

AF — Chronic Atrial Fibrillation; NYHA — New York Heart Association Functional Classiﬁcation III or IV; LVEF — Left Ventricular Ejection Fraction; MVr — Mitral Valve Repair; MVR — Mitral Valve Replacement; NR — Not Reported

*Data are presented as the mean, median (interquartile range).

Table S3. Quality ratings for the cohort studies included on the basis of Newcastle-Ottawa quality assessment scale

|  | Selection  (score) |  |  |  | Comparability  (score) | Outcome  (score) |  |  | Total Score |
| --- | --- | --- | --- | --- | --- | --- | --- | --- | --- |
|  | Representative of exposed cohort | Selections of non exposed cohort | Assessment of exposure | Absence of outcome at start of study | Control for Age or Gender or NYHA class | Assessment of outcome | Follow-up period( >10years ) | Adequacy of  follow-up |  |
| Bozbuga et al[1] | 1 | 1 | 1 | 1 | 2 | 1 | 0 | 0 | 7 |
| Coutinho et al[2] | 1 | 1 | 1 | 1 | 1 | 1 | 1 | 0 | 7 |
| DiGregorio et al[3] | 1 | 1 | 1 | 1 | 2 | 1 | 0 | 1 | 8 |
| Gillinov et al[4] | 1 | 1 | 1 | 1 | 1 | 1 | 1 | 1 | 8 |
| Gramalia et al[5] | 1 | 1 | 1 | 1 | 2 | 1 | 0 | 0 | 7 |
| Grapsa et al[6] | 1 | 1 | 1 | 1 | 1 | 0 | 0 | 0 | 4 |
| Helder et al[7] | 1 | 1 | 1 | 1 | 2 | 1 | 1 | 1 | 9 |
| Hong et al[8] | 1 | 1 | 1 | 1 | 1 | 1 | 1 | 0 | 7 |
| Lazam et al[9] | 1 | 1 | 1 | 1 | 2 | 1 | 1 | 1 | 9 |
| Lee et al[10] | 1 | 1 | 1 | 1 | 1 | 1 | 1 | 1 | 8 |
| Mohty et al[11] | 1 | 1 | 1 | 1 | 0 | 1 | 1 | 1 | 7 |
| Pandis et al[12] | 1 | 1 | 1 | 1 | 0 | 1 | 0 | 0 | 5 |
| Shaﬁi et al[13] | 1 | 1 | 1 | 0 | 0 | 1 | 1 | 0 | 5 |
| Sénéchal et al[14] | 1 | 1 | 1 | 1 | 0 | 1 | 0 | 1 | 6 |
| Tourneau et al[15] | 1 | 1 | 1 | 1 | 2 | 1 | 0 | 1 | 8 |
| Vassileva et al[16] | 1 | 1 | 1 | 1 | 2 | 1 | 0 | 1 | 8 |
| Zalaquett et al[17] | 1 | 1 | 1 | 1 | 1 | 1 | 1 | 1 | 8 |
| Zhou et al[18] | 1 | 1 | 1 | 1 | 1 | 1 | 1 | 1 | 8 |
| Hendrix et al[19] | 1 | 1 | 1 | 1 | 2 | 1 | 0 | 1 | 8 |
| Hata et al [20] | 1 | 1 | 1 | 1 | 2 | 1 | 0 | 1 | 8 |

Reference

1. Bozbuga N, Erentug V, Kirali K, Akinci E, Yakut C. Surgical management of mitral regurgitation in patients with Marfan syndrome. J Heart Valve Dis. 2003;12(6):717-21. Epub 2003/12/09. PubMed PMID: 14658812.

2. Coutinho GF, Correia PM, Branco C, Antunes MJ. Long-term results of mitral valve surgery for degenerative anterior leaflet or bileaflet prolapse: analysis of negative factors for repair, early and late failures, and survival. Eur J Cardiothorac Surg. 2016;50(1):66-74. Epub 2016/01/23. doi: 10.1093/ejcts/ezv470. PubMed PMID: 26792923.

3. DiGregorio V, Zehr KJ, Orszulak TA, Mullany CJ, Daly RC, Dearani JA, et al. Results of mitral surgery in octogenarians with isolated nonrheumatic mitral regurgitation. Ann Thorac Surg. 2004;78(3):807-13; discussion 13-4. Epub 2004/09/01. doi: 10.1016/j.athoracsur.2004.03.041. PubMed PMID: 15336996.

4. Gillinov AM, Faber C, Houghtaling PL, Blackstone EH, Lam BK, Diaz R, et al. Repair versus replacement for degenerative mitral valve disease with coexisting ischemic heart disease. J Thorac Cardiovasc Surg. 2003;125(6):1350-62. Epub 2003/06/28. PubMed PMID: 12830055.

5. Gramaglia B, Imazio M, Checco L, Villani M, Morea M, Di Summa M, et al. Mitral valve prolapse. Comparison between valvular repair and replacement in severe mitral regurgitation. J Cardiovasc Surg (Torino). 1999;40(1):93-9. Epub 1999/04/30. PubMed PMID: 10221393.

6. Grapsa J, Dawson D, Pandis D, Ntalarizou E, Cheung WS, Efthimiadis I, et al. Mitral valve repair results in better right ventricular remodelling than valve replacement for degenerative mitral regurgitation: a three-dimensional echocardiographic study. Hellenic J Cardiol. 2012;53(4):279-86. Epub 2012/07/17. PubMed PMID: 22796815.

7. Helder MR, Schaff HV, Dearani JA, Li Z, Stulak JM, Suri RM, et al. Management of mitral regurgitation in Marfan syndrome: Outcomes of valve repair versus replacement and comparison with myxomatous mitral valve disease. J Thorac Cardiovasc Surg. 2014;148(3):1020-4; discussion 4. Epub 2014/08/19. doi: 10.1016/j.jtcvs.2014.06.046. PubMed PMID: 25129593.

8. Hong JH, Schaff HV, Nishimura RA, Abel MD, Dearani JA, Li Z, et al. Mitral Regurgitation in Patients With Hypertrophic Obstructive Cardiomyopathy: Implications for Concomitant Valve Procedures. J Am Coll Cardiol. 2016;68(14):1497-504. Epub 2016/10/01. doi: 10.1016/j.jacc.2016.07.735. PubMed PMID: 27687190.

9. Lazam S, Vanoverschelde JL, Tribouilloy C, Grigioni F, Suri RM, Avierinos JF, et al. Twenty-Year Outcome After Mitral Repair Versus Replacement for Severe Degenerative Mitral Regurgitation: Analysis of a Large, Prospective, Multicenter, International Registry. Circulation. 2017;135(5):410-22. Epub 2016/12/03. doi: 10.1161/CIRCULATIONAHA.116.023340. PubMed PMID: 27899396.

10. Lee EM, Shapiro LM, Wells FC. Superiority of mitral valve repair in surgery for degenerative mitral regurgitation. Eur Heart J. 1997;18(4):655-63. Epub 1997/04/01. PubMed PMID: 9129898.

11. Mohty D, Orszulak TA, Schaff HV, Avierinos JF, Tajik JA, Enriquez-Sarano M. Very long-term survival and durability of mitral valve repair for mitral valve prolapse. Circulation. 2001;104(12 Suppl 1):I1-I7. Epub 2001/09/25. PubMed PMID: 11568020.

12. Pandis D, Grapsa J, Athanasiou T, Punjabi P, Nihoyannopoulos P. Left ventricular remodeling and mitral valve surgery: prospective study with real-time 3-dimensional echocardiography and speckle tracking. J Thorac Cardiovasc Surg. 2011;142(3):641-9. Epub 2011/02/01. doi: 10.1016/j.jtcvs.2010.11.030. PubMed PMID: 21277588.

13. Shafii AE, Gillinov AM, Mihaljevic T, Stewart W, Batizy LH, Blackstone EH. Changes in left ventricular morphology and function after mitral valve surgery. Am J Cardiol. 2012;110(3):403-8 e3. Epub 2012/04/27. doi: 10.1016/j.amjcard.2012.03.041. PubMed PMID: 22534055; PubMed Central PMCID: PMCPMC4717321.

14. Senechal M, MacHaalany J, Bertrand OF, O'Connor K, Parenteau J, Dubois-Senechal IN, et al. Predictors of left ventricular remodeling after surgical repair or replacement for pure severe mitral regurgitation caused by leaflet prolapse. Am J Cardiol. 2013;112(4):567-73. Epub 2013/05/21. doi: 10.1016/j.amjcard.2013.04.024. PubMed PMID: 23683949.

15. Le Tourneau T, de Groote P, Millaire A, Foucher C, Savoye C, Pigny P, et al. Effect of mitral valve surgery on exercise capacity, ventricular ejection fraction and neurohormonal activation in patients with severe mitral regurgitation. J Am Coll Cardiol. 2000;36(7):2263-9. Epub 2000/12/29. PubMed PMID: 11127471.

16. Vassileva CM, Boley T, Markwell S, Hazelrigg S. Mitral valve repair is underused in patients with hypertrophic obstructive cardiomyopathy. Heart Surg Forum. 2011;14(6):E376-9. Epub 2011/12/15. doi: 10.1532/HSF98.20111067. PubMed PMID: 22167765.

17. Zalaquett R, Scheu M, Campla C, Moran S, Irarrazaval MJ, Becker P, et al. [Long-term results of repair versus replacement for degenerative mitral valve regurgitation]. Rev Med Chil. 2005;133(10):1139-46. Epub 2005/12/13. doi: /S0034-98872005001000002. PubMed PMID: 16341364.

18. Zhou YX, Leobon B, Berthoumieu P, Roux D, Glock Y, Mei YQ, et al. Long-term outcomes following repair or replacement in degenerative mitral valve disease. Thorac Cardiovasc Surg. 2010;58(7):415-21. Epub 2010/10/06. doi: 10.1055/s-0029-1240925. PubMed PMID: 20922625.

19. Hendrix RJ, Bello RA, Flahive JM, Kakouros N, Aurigemma GP, Keaney JF, et al. Mitral Valve Repair Versus Replacement in Elderly With Degenerative Disease: Analysis of the STS Adult Cardiac Surgery Database. Ann Thorac Surg. 2019;107(3):747-53. Epub 2019/01/08. doi: 10.1016/j.athoracsur.2018.09.018. PubMed PMID: 30612990.

20. Hata M, Zittermann A, Hakim-Meibodi K, Borgermann J, Gummert J. Minimally invasive mitral valve repair or replacement for degenerative mitral regurgitation. Interact Cardiovasc Thorac Surg. 2019;28(4):575-80. Epub 2018/11/27. doi: 10.1093/icvts/ivy305. PubMed PMID: 30476075.
