# Supplementary figures and images for: Outcome of mitral valve repair or replacement for non-ischemic mitral regurgitation: a systematic review and meta-analysis
Source: J Cardiothorac Surg. 2021 Jun 15;16:175. doi: 10.1186/s13019-021-01563-2 (PMC8207733; doi:10.1186/s13019-021-01563-2)

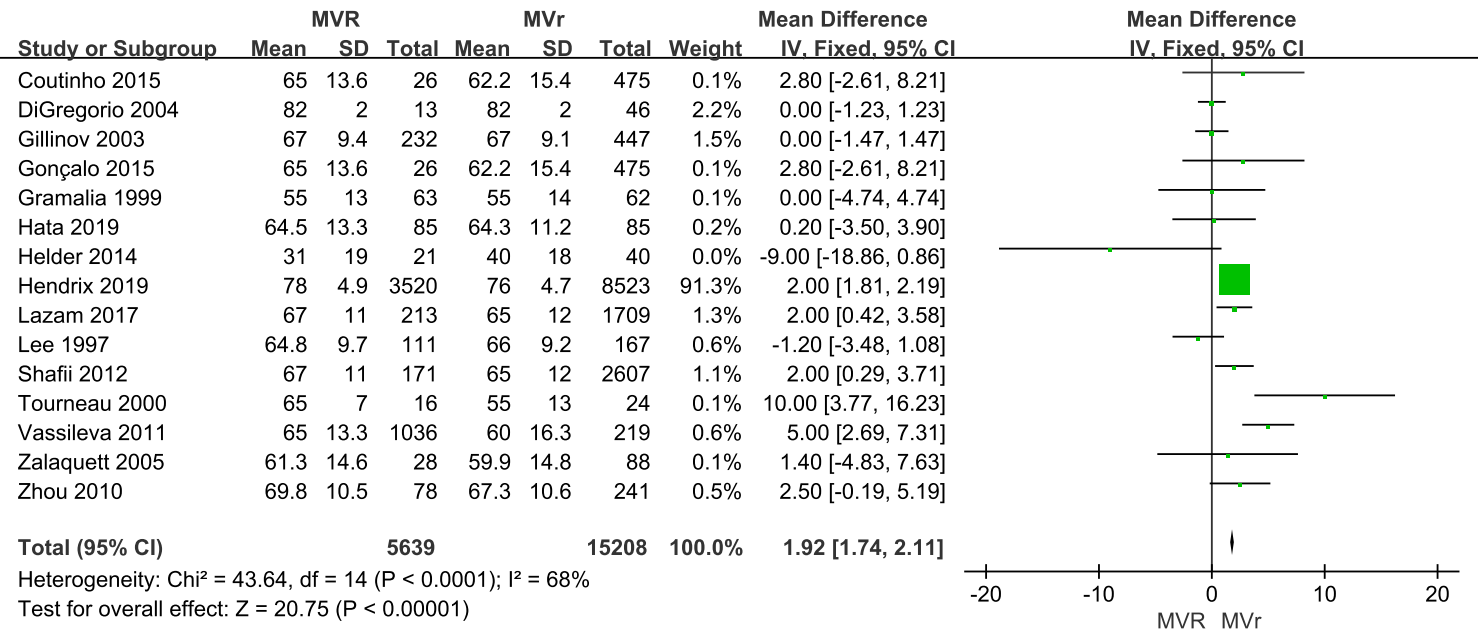

Supplement: Supplementary file 2 — Additional file 2. [file 13019_2021_1563_MOESM2_ESM.pdf]

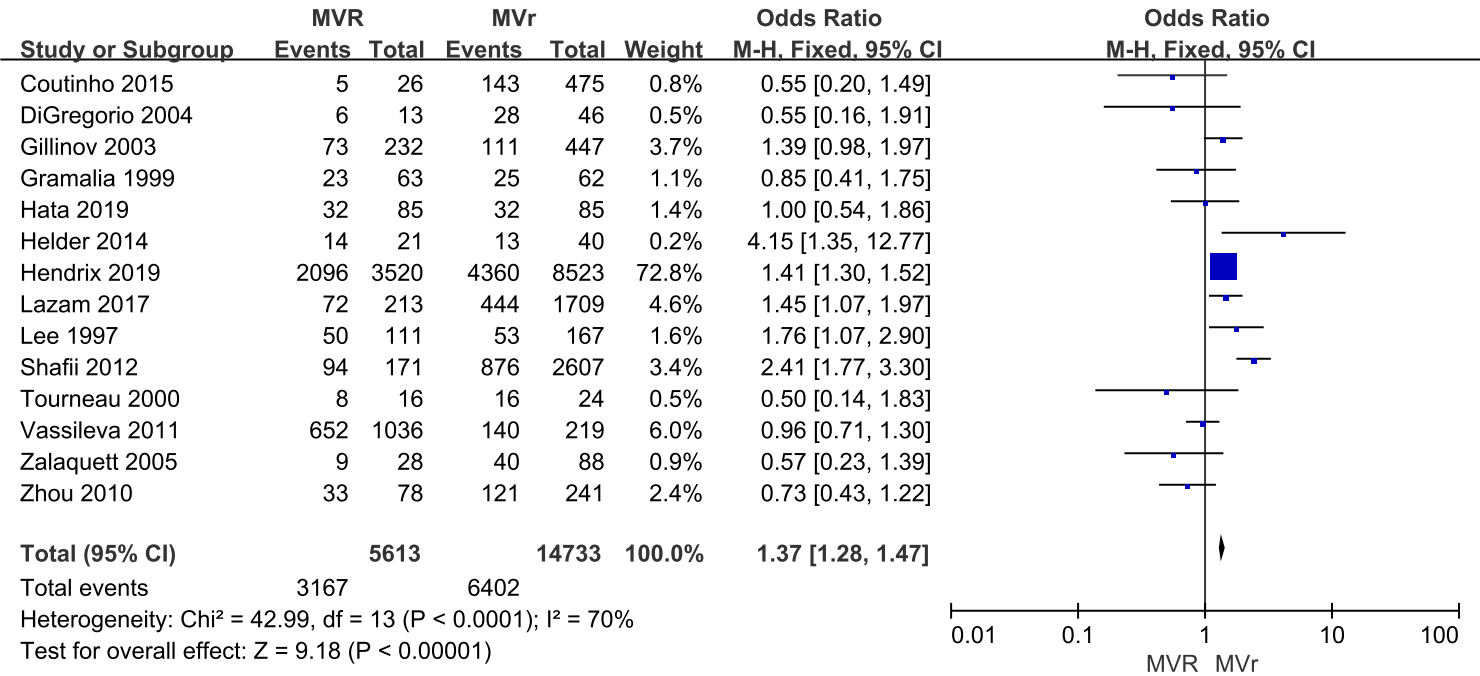

Supplement: Supplementary file 3 — Additional file 3. [file 13019_2021_1563_MOESM3_ESM.pdf]

# A

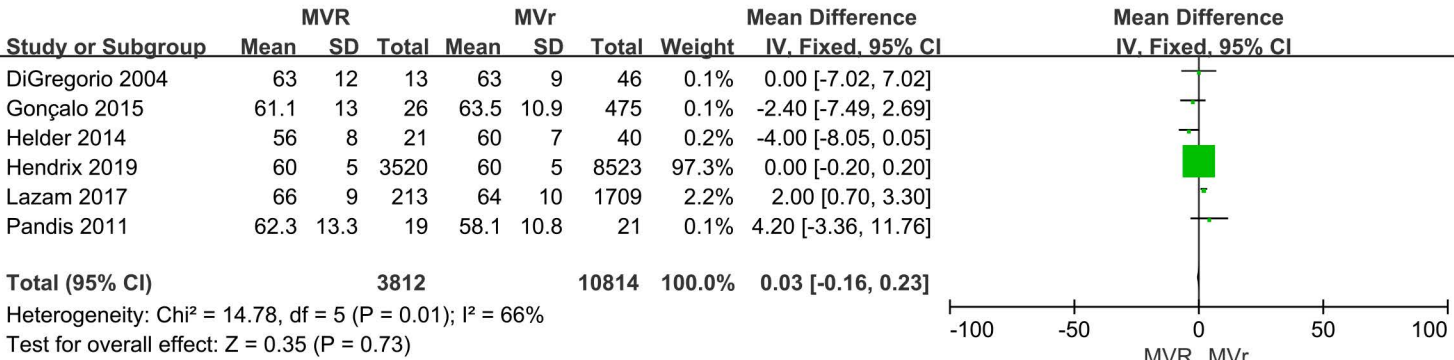

# B

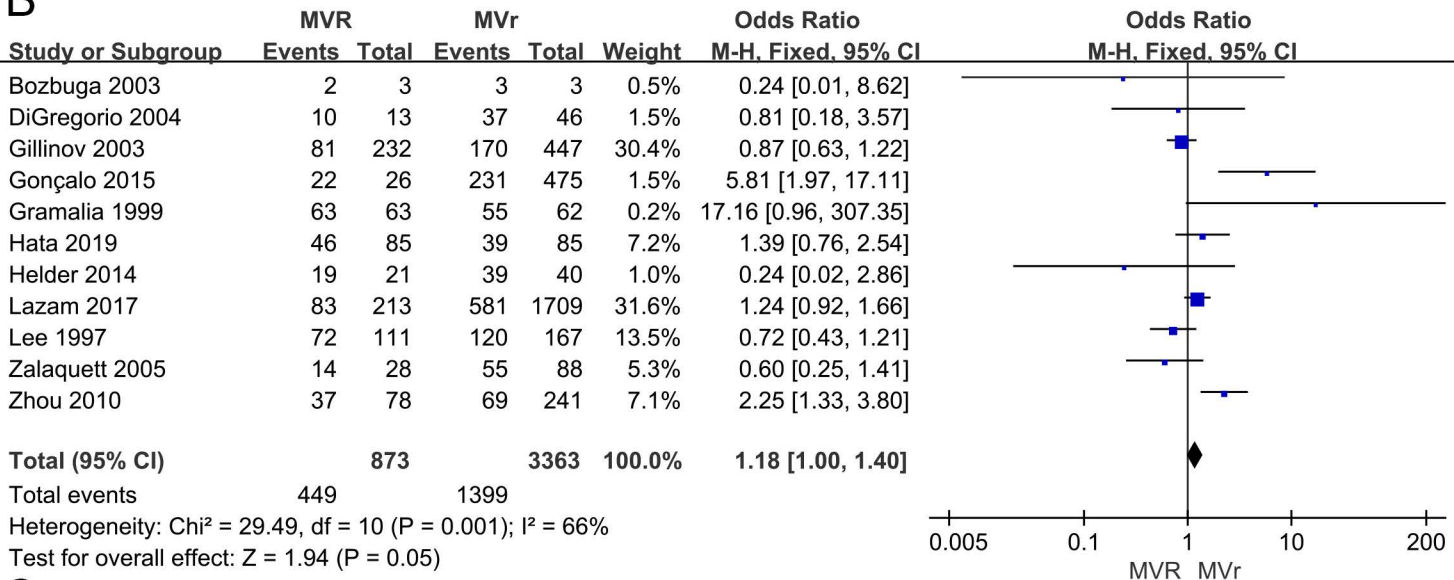

# C

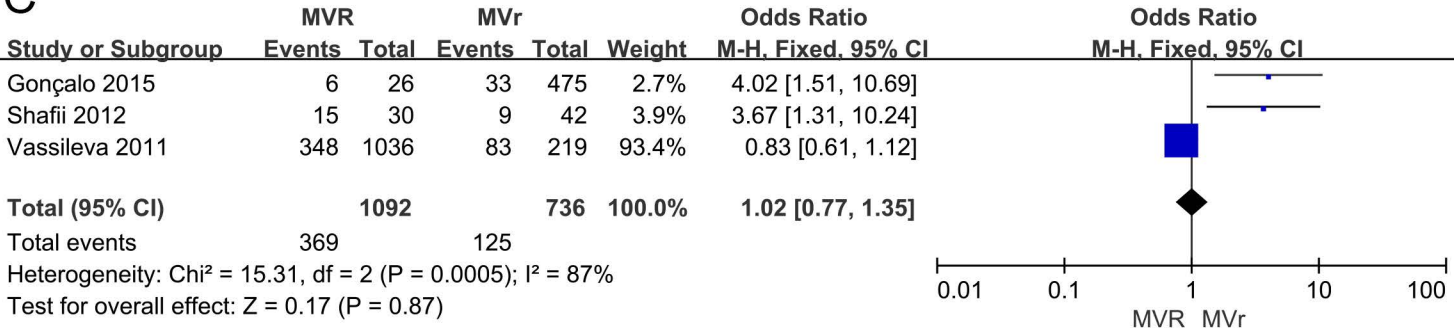

Supplement: Supplementary file 4 — Additional file 4. [file 13019_2021_1563_MOESM4_ESM.pdf]

A

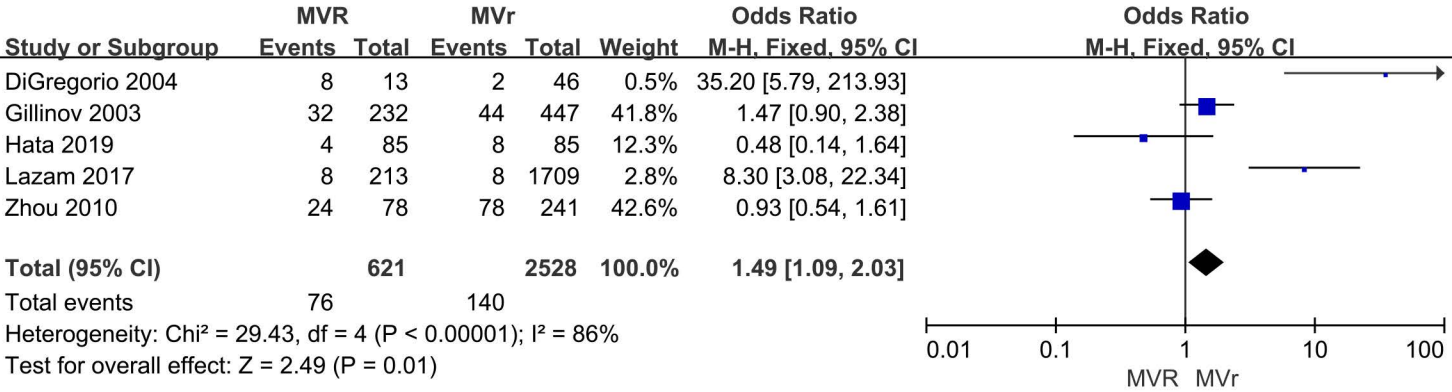

B

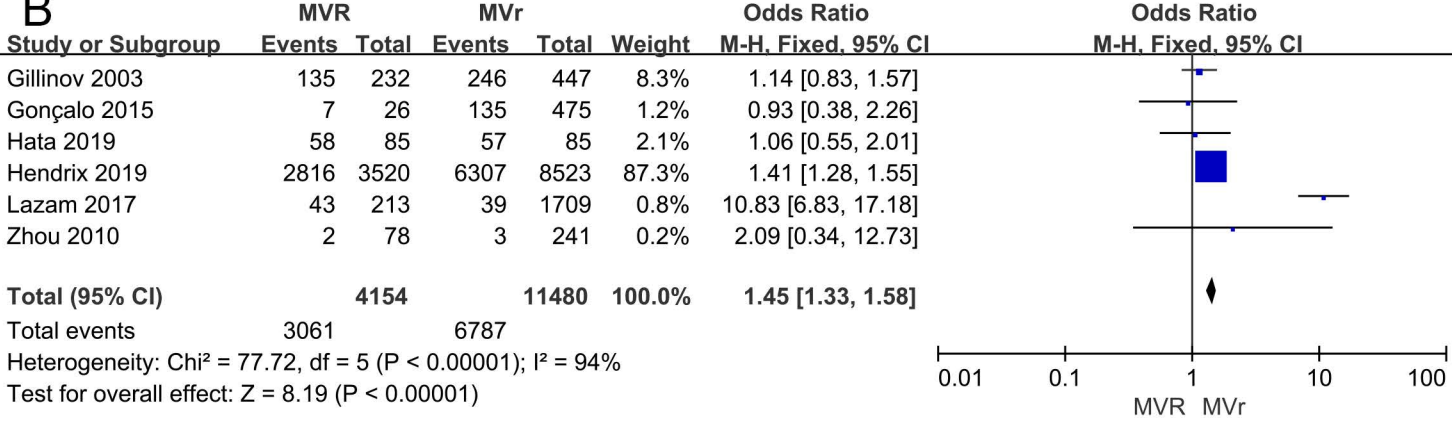

C

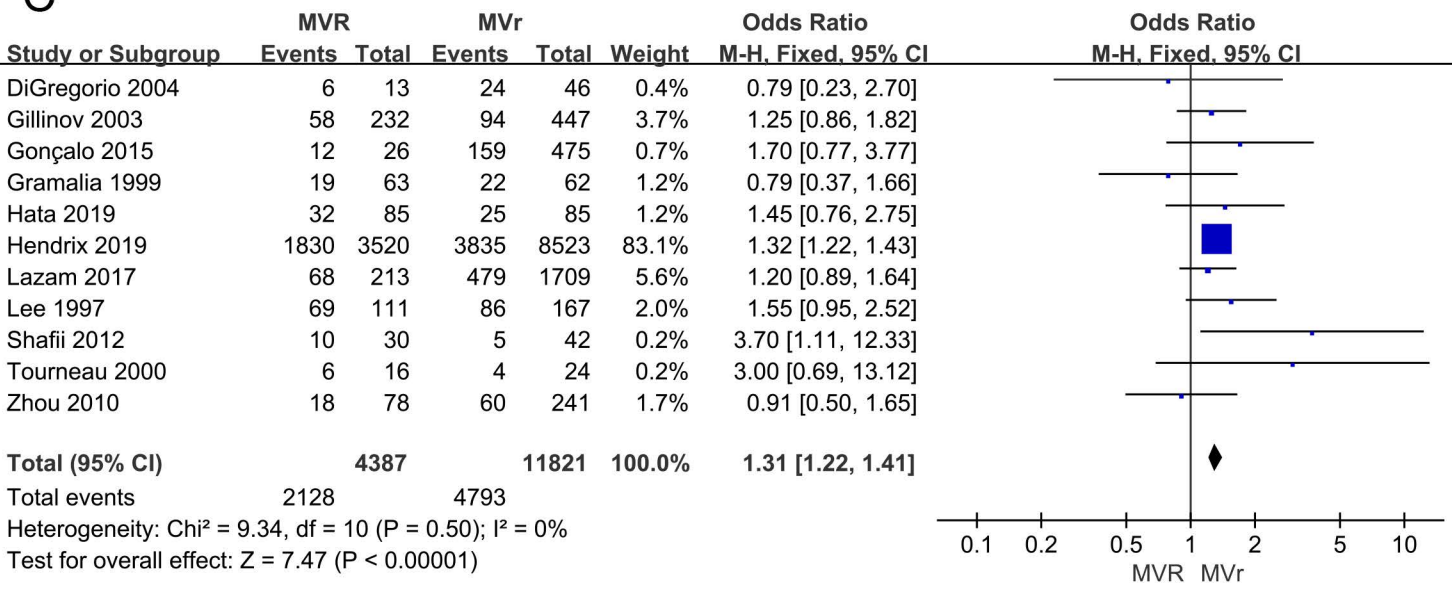

Supplement: Supplementary file 5 — Additional file 5. [file 13019_2021_1563_MOESM5_ESM.pdf]

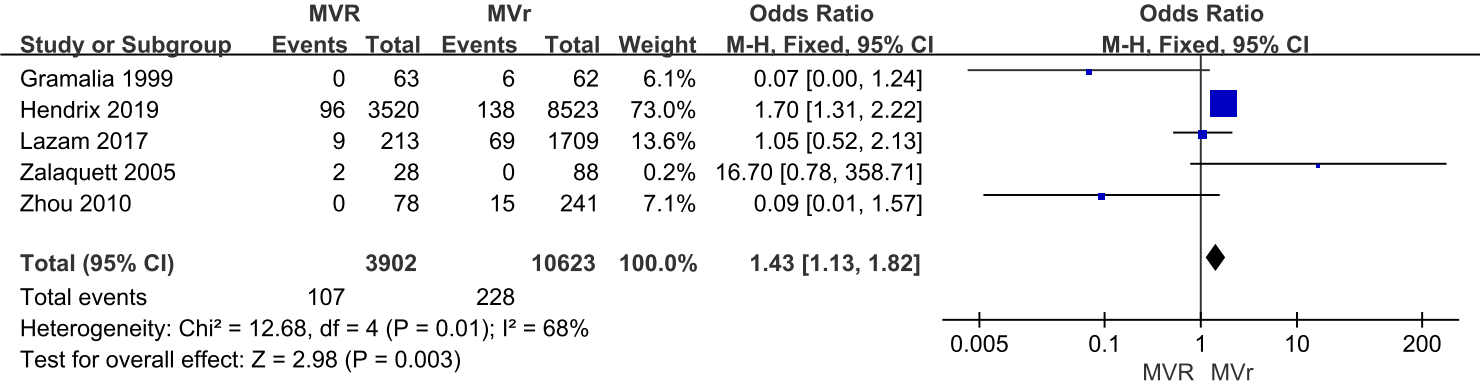

Supplement: Supplementary file 6 — Additional file 6. [file 13019_2021_1563_MOESM6_ESM.pdf]
